# Effect of CNT/PDMS Nanocomposites on the Dynamics of Pioneer Bacterial Communities in the Natural Biofilms of Seawater

**DOI:** 10.3390/ma11060902

**Published:** 2018-05-28

**Authors:** Yubin Ji, Yuan Sun, Yanhe Lang, Lei Wang, Bing Liu, Zhizhou Zhang

**Affiliations:** 1 School of Science, Harbin University of Commerce, Harbin 150076, China; yunbinji@sina.com (Y.J.); sunyuan.2010@163.com (Y.S.); bingliu2018@sina.com (B.L.); 2 Key Laboratory of Saline-alkali Vegetation Ecology Restoration in Oil Field (SAVER), Ministry of Education, Alkali Soil Natural Environmental Science Center (ASNESC), Northeast Forestry University, Harbin 150040, China; langyanhe@163.com; 3 School of Chemistry and Chemical Engineering, Harbin Institute of Technology, Harbin 150001, China; 4 School of Marine Science and Technology, Harbin Institute of Technology at Weihai, Weihai 264209, China

**Keywords:** antifouling coatings, biofouling, natural biofilms, single-stranded conformation polymorphism, polydimethylsiloxane, multidimensional scale analysis

## Abstract

In this study, the antifouling (AF) performance of different carbon nanotubes (CNTs)-modified polydimethylsiloxane (PDMS) nanocomposites (PCs) was examined directly in the natural seawater, and further analyzed using the Multidimensional Scale Analyses (MDS) method. The early-adherent bacterial communities in the natural biofilms adhering to different PC surfaces were investigated using the single-stranded conformation polymorphism (SSCP) technique. The PCs demonstrated differences and reinforced AF properties in the field, and they were prone to clustering according to the discrepancies within different CNT fillers. Furthermore, most PC surfaces only demonstrated weak modulating effects on the biological colonization and successional process of the early bacterial communities in natural biofilms, indicating that the presence of the early colonized prokaryotic microbes would be one of the primary causes of colonization and deterioration of the PCs. C6 coating seems to be promising for marine AF applications, since it has a strong perturbation effect on pioneer prokaryotic colonization.

## 1. Introduction

The occurrence of biofouling on synthetic surfaces is a major issue for the shipping industries in marine environments [1], which has resulted in substantial economic and ecological consequences. For example, total cruise expenses are greatly increased by approximately 77% annually worldwide, primarily owing to the constantly enhanced propulsive power and fuel consumption [2]. Natural biofilms, also termed microfouling, are well-organized and complex assemblages, mainly developed by the undesirable colonization of marine microorganisms as well as their extracellular matrix materials [3,4,5]. Over the past few decades, the early-adherent biofilm-forming marine bacteria communities on the artificial surfaces aroused researchers’ interests worldwide, since their presence was found to be closely related to the subsequent macrofouling process, which can further enhance the potential hazards of the biodeterioration and biodegradation of the selected biofouling-resistant substrata, thereby leading to a remarkable loss in antifouling (AF) performances [6,7,8].

So far, the most commonly used remedial strategies to retard the build-up of biofouling have taken the form of protective coatings, broadly categorized into AF and fouling-release (FR) coatings [9,10]. Traditionally, biocide-released AF coatings have been demonstrated to be environmentally damaging to non-target living marine organisms, due to the presence of a range of poisonous organic biocides, such as tributyltin (TBT). Therefore, their use in the coating industry has been globally restricted and prohibited [11]. As a consequence, the search for favorable biocide-independent coatings for biofouling management has been greatly accelerated [12], particularly in regard to FR coatings [13].

The organo–silicon polymers, typically the polydimethylsiloxane (PDMS), represent a desired non-toxic alternative and marked niche among specialty copolymers [14]. The PDMS resin possesses a superior environmentally-friendly nature, with characteristics such as high heat resistance, surface inertness, high hydrophobicity, as well as excellent fouling anti-adhesion characteristics, presenting viable options in several marine industries [15]. Furthermore, these PDMS-based nanocomposites have been systematically investigated in recent years, mainly because of their facile preparation and ecological stability [16,17]. Many research studies and testing procedures have been devoted to meeting the challenge of exploring effective, reliable and high-performance inorganic nanofillers, for the purpose of obtaining PDMS-based nanocomposites with reinforced AF and FR properties [18]. Carbon nanotubes (CNTs) are considered one of the most favorable inorganic fillers for PDMS modification [19], while the PDMS nanocomposites (PCs) seem to be the most promising candidate for marine anti-biofouling applications, although the potential impact of CNTs on the biological colonization dynamics of the early biofilm-forming bacterial communities still remains poorly understood. In addition, the culture-independent molecular fingerprinting method, i.e., the single-stranded conformation polymorphism (SSCP) technique, has been widely used to estimate the global diversity of environmental microbial communities in the field of microbial ecology in recent years [20].

Therefore, the current study aimed to investigate the effects of different CNT modified PDMS composites (PCs) on the colonization dynamics of the pioneer bacterial communities in the natural biofilms using the single-strand conformation polymorphism (SSCP) technique. The clustering patterns of the early bacterial biofilm communities adhering to various PCs were explored using Multidimensional Scale Analyses (MDS). In addition, a surface evaluation system based on the MDS method was established in order to quantify the fouling conditions among different PC surfaces examined in the field.

## 2. Materials and Methods

### 2.1. Materials

#### 2.1.1. The Primer Coat

The primer coat, i.e., the chlorinated rubber iron-red antirust paint, was kindly supplied by the Jiamei Company (Weihai, China), and consisted primarily of chlorinated rubber resin, micaceous iron oxide, plasticizers, additives and a mixed solvent. The primer paint was cured for approximately 72 h at room temperature (RT).

#### 2.1.2. Silicone-Based Matrix System

The silicone-based matrix used in this study was, necessarily, PDMS (P0) resin from a Sylgard 184 elastomer kit, purchased from the Dow Corning Company (Shanghai, China). This commercially available PDMS material acted as a standard resin for further preparation processes. The PDMS polymer was obtained directly by mixing the pre-polymer (Component A) to the curing agent (Component B) in a ratio of 10:1 (weight) at 105 °C within 6 h, which served as the standard coating controls.

#### 2.1.3. Carbon Nanotubes (CNTs)

All CNTs used in the current study were purchased from the Chengdu Organic Chemicals Co., Ltd. (Chengdu, China), Chinese Academy of Sciences, including six multi-walled carbon nanotubes (MWCNTs, F1-F6), six hydroxyl-modified MWCNTs (hMWCNTs, F7-F12), and six carboxyl-modified MWNTs (cMWCNTs). Detailed information about these CNTs was summarized as presented in Table 1. The CNTs were incorporated in the PDMS matrix at concentrations of 0 (PDMS only) and 0.1% (*w*/*w*), respectively, as previously reported by Beigbeder and coworkers [21].

#### 2.1.4. Preparation of the PDMS-Based Composites (PCs)

Eighteen kinds of PCs were freshly produced, which were largely cataloged into three sets: the M set (MPs, M1–M6), the H set (HPs, H1–H6) and the C set (CPs, C1–C6). The composition of these PCs are summarized in Table 1. These PCs were all formulated following a similar procedure [20]. Briefly, each CNT filler was blended with base elastomer (Part A) for 10 min by intense stirring at 500 rpm for 1 min. Then, the suspension was well mixed with the curing agents (Part B), and mechanically stirred for another 15 min. The air bubbles from the PDMS mixture were completely removed using a vacuum desiccator. Afterwards, these PCs were cured at 105 °C for 6 h in a constant temperature oven.

### 2.2. Panel Preparation

The steel panels (measuring 10 cm × 10 cm × 3 cm) for the seawater exposure assays were firstly drilled at the bottom, and then thoroughly polished with the abrasive paper of different grits in order to obtain the same surface condition in terms of roughness. Afterwards, these panels were carefully washed with sterile H_2_O and rinsed with 70% (*v*/*v*) ethanol, then dried at room temperature overnight prior to use. A layer of the primer coat was coated on each panel and dried for 72 h at room temperature. Then, these pre-treated panels were coated with the PCs using a bar-coater and cured for 6 h at 105 °C in an oven. A minimum of 3 specimens of each PC was produced for further statistical evaluation.

### 2.3. Seawater Exposure Assays

**Figure 1:**
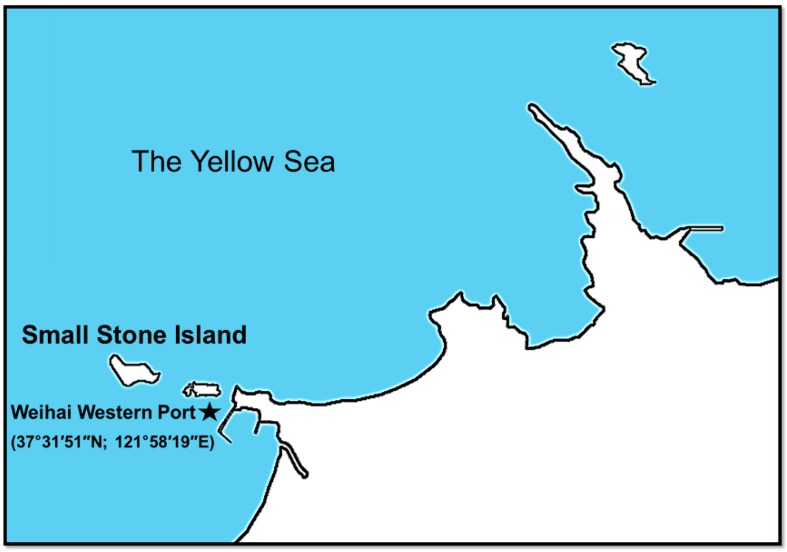
Location of the immersion sites for the field studies: Small Stone Island in the Western Port, Weihai, China.

The field exposure studies were performed at a static woody pontoon in a marina named Small Stone Island in the Weihai Western Port, China (37°31′51′′ N; 121°58′19′′ E, see Figure 1). Panels coated with different experimental materials were produced in triplicate throughout. These panels were randomly arranged on a wooden pontoon located in the Small Stone Island harbor waters using thin ropes, and then vertically suspended at 1.5 m below the lowest tide level over a period of 56 days (April–June, 2015). The average sea temperature during the exposures was 11 °C throughout.

The fouling conditions of each experimental material were captured using a digital camera from the fourth week after immersion at one-week intervals, namely at 28 days, 35 days, 42 days, 49 days and 56 days. After photographing, these panels were sent back to the marine realms as quickly as possible. According to the captured images, fouling conditions were further quantified according to the amount of adherence of the major fouling organisms, including barnacles (*B. Amphitrite*), mussels (*Mytilus edulis*), *Ulva pertusa*, sessile ascidian, as well as seaweeds. It is notable that the aforementioned scoring procedures were conducted at five different exposure times, i.e., each experimental material amounted to scoring fifteen times, since each experimental material was prepared in triplicate. Furthermore, owing to the edge effects, the 20 mm area from the margin of each tested panel was excluded within the scope of the assessment area. The clustering patterns of the AF properties of different PDMS-based coatings were performed by inputting the substratum and assessment outcomes as variables using the MDS method conducted by SPSS19.0 software (IBM, Armonk, NY, USA). The pure PDMS coated panels served as standards.

### 2.4. Sampling

The short-term in situ experiments were performed at the same field immersion sites, using the PDMS-based coatings as the artificial substrata for the biofilm recruitments. The formation of biofilm on each PDMS-based coating surface was measured throughout the two-week in situ experiment (April 2–15, 2015) at five different points in time: April 3 (2-day biofilm), April 6 (5-day biofilm), April 9 (8-day biofilm), April 12 (11-day biofilm), and April 15 (14-day biofilm). For each PDMS-based coating, a replicate of four panels (measuring 10 cm × 10 cm) was prepared throughout the investigation. For each panel, an area of approximately 80 mm × 80 mm within each PC surface was sampled.

All tested steel panels were brought back to the laboratory as quickly as possible using a cool-box. Each panel was carefully rinsed with the sterile artificial seawater prior to scrapping, in order to remove the excess sediment and temporarily attached microorganisms. The biofilm samples were gently scraped from the surfaces of each tested panel using the sterile brushes. The replicated scrapings belonging to the same PCs were collected into a sterile Eppendorf tube (2.0 mL) as a representative of all replicate biofilm samples for the subsequent microbial assays. Afterwards, the biofilm samples were suspended into 400 μL sterile deionized water, and vortexed for 60 min prior to being centrifuged, aiming to pellet the biomass at 4000 rpm for 5 min. Then, these biofilm pellets were stored at −80 °C for further analysis.

### 2.5. SSCP

The genomic DNA extractions were performed on all biofilm samples using the Sangon Rapid Bacterial Genomic DNA Isolation kit (Cat# B518225). The integrity of the genomic DNA was examined by 0.8% agarose gel electrophoresis and further quantified by determining the absorbance at 260 nm. Amplification of the prokaryotic 16SrRNA gene fragments was undertaken using the general primer pairs synthesized from Sangon (Shanghai, China), namely 337F (5′-GAC TCC TAC GGG AGG CWG CAG-3′) and 1100R (5′-GGG TTG CGC TCG TTG-3′), which were used to identify the early prokaryotic microbes in the pioneer natural biofilms formed on different PC surfaces, yielding a fragment of ~763 bp. The asymmetric PCR amplification of the target 16S RNA gene fragments was conducted following similar procedures as described previously [22]. A negative control was included throughout. Afterwards, the PCR products were detected by 1.5% agarose gel electrophoresis and stored at −40 °C for further analysis.

The SSCP analysis was performed on a DYCZ-24DN vertical gel electrophoresis apparatus (Liuyi, Beijing). All 16S rDNA fragments were well blended with equal volumes of the denaturation solution separately, which contained 95% formamide, 0.25% bromphenol blue and 0.25% xylene cyanol. The PCR products were denatured at 98 °C for 10 min, and then snap-frozen on ice prior to loading. Then, these denatured PCR products (6.0 μL) were loaded onto 8% (*w*/*v*) polyacrylamide (arylamide:bisacrylamide = 29:1) gel with a thickness of 1 mm, and separated at a constant voltage of 90 V for 28 h in 1× TBE buffer at 4 °C. Subsequently, the SSCP gels were silver stained. A digitized image of the SSCP gels was captured using a digital camera, and the lanes and bands in the SSCP gel images were further analyzed using the Quantity One analysis software (Bio-Rad, Hercules, CA, USA), according to the position of each nucleic acid band, thereby resulting in a matrix based on the presence/absence of bands.

### 2.6. Data Analysis

The SSCP presence/absence binary data matrices constructed in terms of band positions and intensities were used to identify the differences between the pioneer bacterial communities developed on the pure PDMS and CNT modified PDMS composites via the comparison of the diversity indices calculated by the Biodap software, which were able to give detailed descriptions of the dynamics of the early bacterial communities. The clustering analysis was done for the pioneer prokaryotic communities on different PDMS-based composites based on MDS using the SPSS19.0 software package (IBM, Armonk, NY, USA), primarily performed with substratum and diversity indices as variants. Furthermore, the statistical differences between the diversity indices were compared using *t* tests (*p*-value < 0.05, GraphPad Prism 6.0).

## 3. Results and Discussion

### 3.1. Fouling and Surface Evaluation

**Figure 2:**
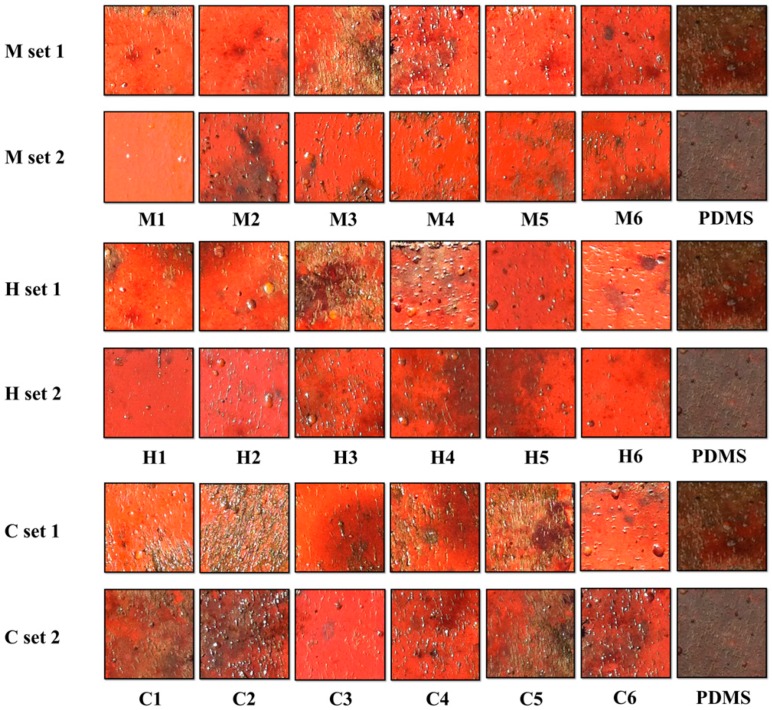
Appearances of various PDMS-based panels after static immersion for two months (April–June, 2015).

**Figure 3:**
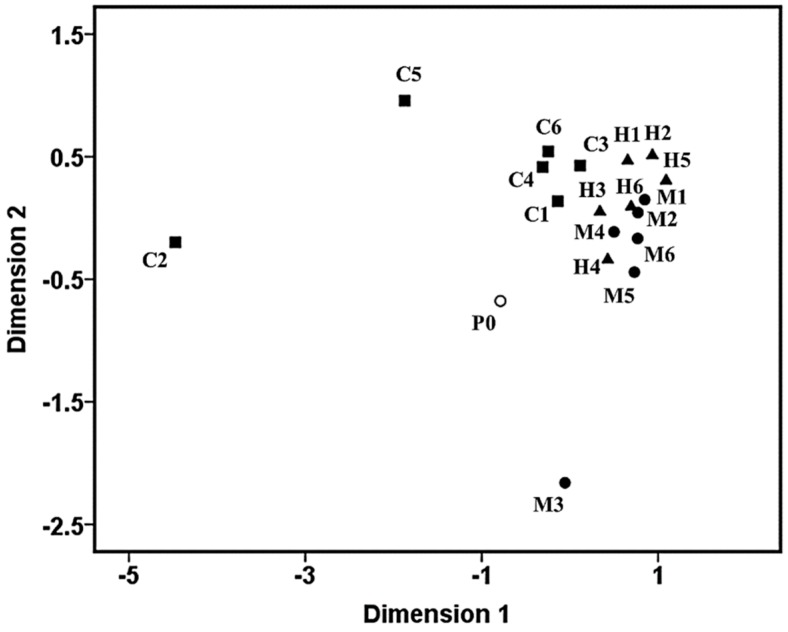
Clustering patterns of the antifouling (AF) capacity of the PDMS-based nanocomposites based on the MDS analysis.

In this study, we directly examined the AF capacity of the aforementioned PDMS-based coatings in natural seawater and established a fouling evaluation system based on the MDS method in order to further quantify the fouling conditions among different PC surfaces, in the context of the adhesive number of five representative major macrofoulers, including barnacles, mussels, ascidians, *Ulva* and seaweeds (see Figure 2 and Figure 3).

From Figure 2, it is clear that the AF properties of the plain PDMS were greatly improved after the incorporation of a low amount of nanosized CNTs (0.1 wt %). Each PC displayed a differential but reinforced AF efficacy against the representative macrofoulers compared with the PDMS standard. The M1, H1 and C3 coatings performed exceptionally well in the field exposure assays, while the M3, C2 and C5 coatings were found to be heavily fouled. Furthermore, it seems that most PCs of the identical set (e.g., coatings in the M Set, except M3) tended to exhibit similar AF properties, although there were some exceptions (e.g., C2 and C5 coatings in the C Set). These differential AF behaviors may be largely owing to the differences within different CNT fillers.

In addition, in Figure 3, most PCs, including M1, M2 and M4-M6 coatings from the M set, H1–H6 coatings from the H set, and C1, C3, C4 and C6 coatings from the C set, were liable to cluster into the same group, suggesting that these PCs may have possessed similar AF performances. Nevertheless, it is noticeable that M3, C2 and C5 coatings were liable to cluster separately, and their AF properties were clearly different from those of the PDMS standards (P0) and the aforementioned PCs. This result further revealed that the physicochemical properties of the CNT filler may have differential reinforcing impacts on the AF properties of the PDMS matrix. Recently, CNTs have been applied as additives to improve the membrane properties of various polymeric matrixes worldwide, and a host of highly promising functionalized nanocomposites with excellent properties have been obtained for marine AF applications [23,24]. However, most fouling evaluation systems are still confined to laboratory assays, only involving the measurement of the adhesive number of representative hard foulings (e.g., *B. Amphitrite* and *Mytilus edulis*) [25,26] or soft foulings (e.g., *Ulva*) [27,28]. It is obvious that laboratory biological assays still remain insufficient and limited, although laboratory assays are insusceptible to environmental disturbances, unlike field exposure assays [29]. Here, we provided a feasible and effective way to solve this problem and established a novel fouling evaluation system targeting the measurement of the adhesive number of multiple natural fouling organisms in natural seawater using the MDS method, based on the data obtained from rigorous marine field assays. The advantage of this approach is obvious, since the adhesive behaviors of multiple adherent marcofoulers on different coating surfaces can be dynamically observed and recorded directly in the natural seawater, which can give a more comprehensive and objective assessment on their actual AF performance. Besides, the variations within different AF coatings can also be easily observed and captured simply using visual inspection.

### 3.2. SSCP Patterns of the Bacterial Biofilm Communities

**Figure 4:**
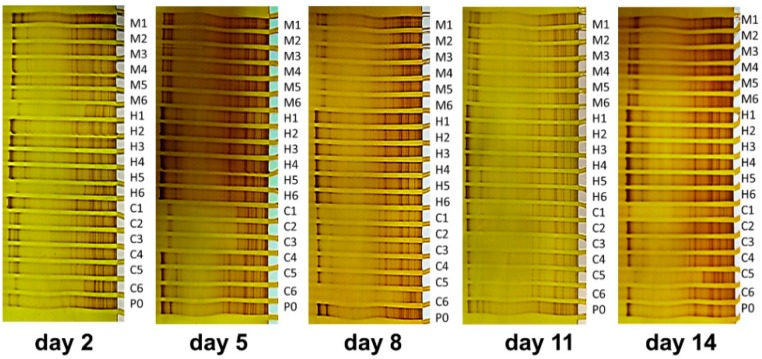
SSCP fingerprints of pioneer bacterial communities in the natural biofilms developed on different PDMS-based composite surfaces with different exposure times.

Figure 4 shows the SSCP profiles of the pioneer bacterial communities in the natural biofilms developed on the PDMS-based material surfaces at different exposure times. Each band within the SSCP profiles is approximately identical to a single microbial species. As observed from SSCP patterns, eighteen kinds of PCs were generally colonized by a mixture of the early-adherent bacterial communities without exception during the two-week in situ experiment, and no significant differences were screened compared with the PDMS standards (P0) via the visual inspection. This indicated that no PCs completely resisted or deterred the colonization of pioneer prokaryotic microbes.

Early bacterial communities formed on the PDMS-based coatings belonging to the same PC set were liable to evolve similar SSCP patterns at different exposure times, while differential SSCP patterns were screened within different PC sets. For example, in the 5-day biofilm, clear differences were observed in the SSCP patterns of the pioneer biofilm communities developed on different PC sets, owing to the differences within various coating types. It is estimated that the physicochemical properties of the CNT types may be closely related to the differential and improved AF properties of the PCs. In addition, as the natural biofilm grew older (e.g., the 14-day biofilm), the early adhered bacterial communities on the PDMS-based coatings were found to be clearly increased. This result suggested that the deterrence effects of PCs against the colonization of the early bacterial communities may become increasingly weakened over time. These combined results indicated that the PCs were susceptible to microfouling when immersed in the marine environment during the short-term in situ experiment.

### 3.3. Clustering Patterns of the Pioneer Bacterial Communities

**Figure 5:**
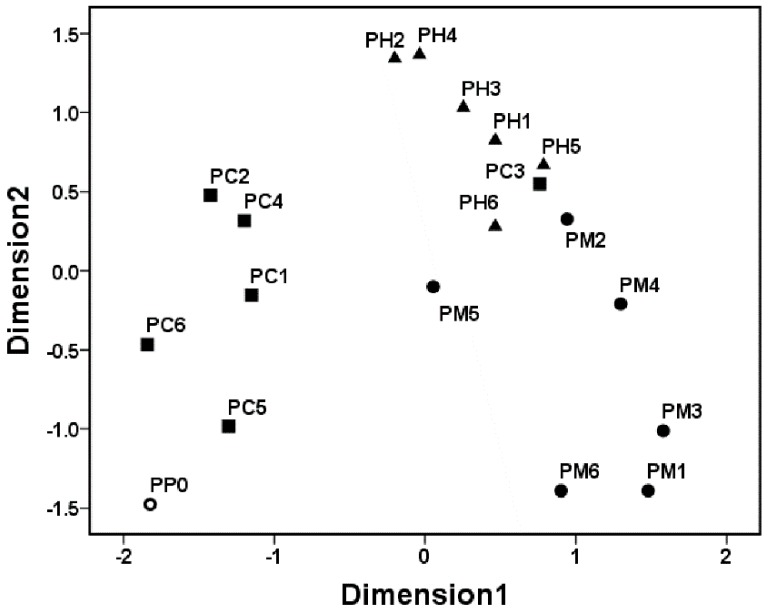
Clustering analysis of pioneer bacterial communities on different PDMS-based material surfaces based on the MDS method. PP0, PM, PH and PC represent the pioneer bacterial communities adhering to the surfaces of P0 coating, M1–M6 coating, coating H1–H6 coating and C1–C6 coating, respectively.

Figure 5 shows the clustering patterns of the pioneer bacterial communities on the PDMS-based coating surfaces using the MDS method. The pioneer bacterial communities developed on different PCs had clear differences from the PDMS standards, indicating that different PCs demonstrate differentially perturbation effects on the colonization of early bacterial communities in natural biofilms. The pioneer bacterial communities were liable to be grouped or clustered on most PC surfaces of the same PCs set (e.g., the M set), while the pioneer bacterial communities adhering to surfaces of different PCs sets were prone to show clear differences. For example, the clustering patterns of the pioneer bacterial communities attached to the MPs surfaces belonging to the M set were quite different from those attached to the CP surfaces belonging to the C set, indicating that the types of PCs may be strongly related to the differences in clustering patterns of the early biofilm communities. However, it is noticeable that the modulating effects of the PCs became gradually weaker with the growth of the natural biofilms, as evidenced by the SSCP analysis (See Figure 4). Furthermore, it seems that the AF properties of the PCs have no necessary relationships with the clustering features of the pioneer bacterial communities, as evidenced by the results shown in Figure 2 and Figure 3, although the pioneer bacterial communities may contribute considerably to the subsequent macrofouling occurring on the surfaces of the PCs.

### 3.4. Analysis of Pioneer Bacterial Communities in the Natural Biofilms

**Figure 6:**
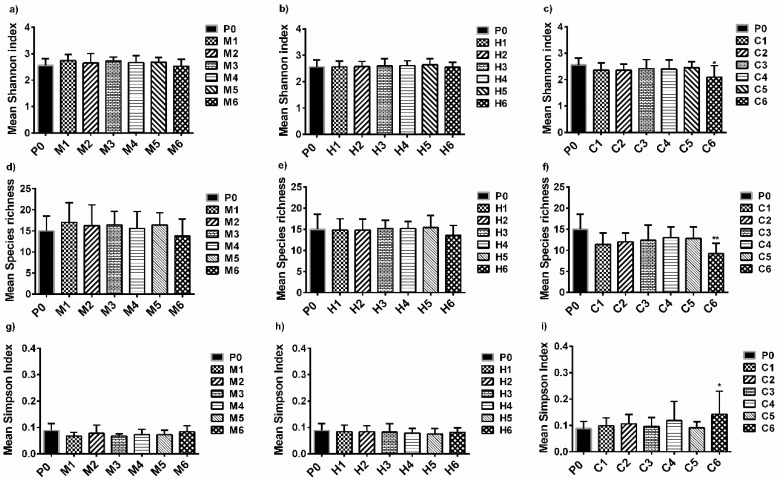
The **comparison** of the diversity indices, (**a**–**c**) Shannon diversity index, (**d**–**f**) species richness, (**g**–**i**) Simpson index of pioneer bacterial communities on different PDMS-based material surfaces.

Three diversity indices, including the Shannon diversity index (*H*), species richness(*S*), and the Simpson index (*λ*), were calculated and compared, as presented in Figure 6.

The Shannon diversity index (*H*) describes the general biodiversity in environmental microbial communities, and was used to estimate the early bacterial community diversity in the natural biofilms developed on different PCs [30]. Figure 6a–c show that the *H* value of the bacterial communities ranged between 2.53 ± 0.27 and 2.73 ± 0.23 for all the PC surfaces, compared with the PDMS control (2.56 ± 0.26), indicating that different PC surfaces may have differential modulating effects on the colonization of pioneer bacterial communities. The highest level of early bacterial community diversity was screened on the M1 surfaces (*H* = 2.73 ± 0.23) among all of the PC surfaces, while the lowest level of diversity was found on the C6 surfaces (*H* = 2.53 ± 0.27). The pioneer prokaryotic microbial communities attached to the PCs surfaces belonging to the M set and *H* set (H1–H6) shared a relatively high level of diversity, with *H* values ranging from 2.53 ± 0.27 to 2.73 ± 0.23 and 2.54 ± 0.19 to 2.64 ± 0.23 (Figure 6a,b), respectively. However, the diversity of the early bacterial communities on the CP surfaces (C1–C6) was lower than that of the PDMS control (2.56 ± 0.26), with *H* values ranging from 2.09 ± 0.44 to 2.45 ± 0.22 (Figure 6c), particularly on the C6 surfaces (*p* < 0.05). No significant differences were found in the diversity level between the PCs (except C6) and the PDMS standards (*p* > 0.05). This indicated that the PC surfaces may only have weak modulating effects on colonized pioneer prokaryotic microbes, and most PC surfaces were still susceptible to the colonization and deterioration of the pioneer prokaryotic microbes.

Furthermore, species richness (S) describes the number of different species in an environmental microbial community, which was applied to give descriptions about the number of species of the early bacterial communities in the natural biofilms developed on different PCs [31]. Figure 6d–f revealed that the S value of the pioneer bacterial community (ranging from 9 ± 2 to 17 ± 5) was slightly downregulated by most PC surfaces (except C6), compared with the PDMS surface (15 ± 4). Specifically, the pioneer bacterial biofilm communities adhering to the PC surfaces belonging to the MPs surfaces and the HPs surfaces shared a relatively high level of richness, while the early bacterial communities adhering to the PCs belonging to the C set shared a relatively low level of richness, significantly on the C6 surfaces (*p* < 0.01), compared to the PDMS control, which correlated well with the diversity level.

The Simpson index (λ) describes the number of dominant species in a particular microbial community and was used to measure the number of dominant populations in pioneer bacterial communities in the natural biofilms formed on different PCs [32]. Figure 6g–i reveal that the *λ* value of pioneer bacterial communities was slightly decreased on the MP surfaces (0.066 ± 0.013 to 0.083 ± 0.023), and remained almost unchanged on the HP surfaces, while it slightly increased on the CP surfaces (0.093 ± 0.021 to 0.160 ± 0.088), particularly on the C6 surfaces (*p* < 0.05), compared to the PDMS control (0.088 ± 0.027). This result indicates that the dominant bacterial population in the biofilm developed on most PCs surfaces (except C6) varied slightly, in contrast to the PDMS control. The slightly changed dominant bacterial communities in the biofilm suggest that most PC surfaces may only have a weak capacity to exert enough perturbations on the biological colonization and successional patterns of early adherent bacterial communities in natural biofilms.

Previously, a host of publications reported that AF coatings could influence and regulate the development of early-colonized bacterial communities [33,34,35,36]. However, a few publications have focused on the modulating effects of different PDMS-based nanocomposites on colonized pioneer bacterial communities in natural biofilms [37,38]. In the current study, most PCs demonstrated differential modulating effects on the colonization of pioneer prokaryotic microbes. The pioneer bacterial communities were only found to be subjected to the minor perturbations exerted by most PCs (except for coating C6). This slightly modulating effect suggested that the PCs may not exert sufficient perturbations on the biological succession patterns of the pioneer bacterial communities in the natural biofilms, which may contribute to the mechanisms causing the plain PDMS surfaces, along with most PCs surfaces (except coating C6), to be extremely susceptible heavy fouling after long-term exposure to the marine environment, since the bacterial communities in the biofilms have been found to play key roles in the biodeterioration and biodegradation of synthetic polymeric materials [39]. Data on this hypothesis still requires further study in our future work. Coating C6 seems to be promising for future marine anti-biofouling applications, owing to its strong perturbation effects on the colonization of early bacterial communities.

## 4. Conclusions

The present study examined the AF capacity of eighteen kinds of PDMS-based composites (PCs) via field exposure assays and provided the first example of quantifying and evaluating their AF efficacy using the MDS method. The bacterial community analysis based on the SSCP fingerprints revealed that most PCs (except C6) have weak modulating effects on the biological colonization of the early-adherent prokaryotic microbes, which may account for the mechanisms of biofouling that occurred on the PDMS-based coating surfaces. This study may lead the way to the development of a number of effective, reliable, and long-lasting ecofriendly coatings for marine anti-biofouling applications.

## Figures and Tables

**Table 1:** The carbon nanotube (CNT) fillers and the polydimethylsiloxane (PDMS) composites (PCs) in the current study.

CNTs	Hydroxyl Content % (*w*/*w*)	Carboxyl Content % (*w*/*w*)	Diameter (nm)	Length (μm)	SSA (m^2^/g)	PC Sets	PC Names
F1	_	_	10–20	30–100	>165	M	M1
F2	_	_	8–15	~50	>233	M	M2
F3	_	_	10–20	10–30	>200	M	M3
F4	_	_	20–30	10–30	>110	M	M4
F5	_	_	30–50	10–20	>60	M	M5
F6	_	_	>50	10–20	>40	M	M6
F7	5.58	_	<8	10–30	>500	H	H1
F8	3.70	_	8–15	~50	>233	H	H2
F9	3.06	_	10–20	10–30	>200	H	H3
F10	1.76	_	20–30	~30	>110	H	H4
F11	1.06	_	30–50	~20	>60	H	H5
F12	0.71	_	>50	~20	>40	H	H6
F13	_	3.86	<8	~30	>500	C	C1
F14	_	2.56	8–15	~50	>233	C	C2
F15	_	2.00	10–20	10–30	>200	C	C3
F18	_	1.23	20–30	~30	>110	C	C4
F17	_	0.73	30–50	~20	>60	C	C5
F18	_	0.64	>50	~20	>40	C	C6

Note: F1–F6, F7–F12 and F13–F18 represent different types of multi-walled carbon nanotubes (MWCNTs), hydroxyl-modified MWCNTs (hMWCNTs) and carboxyl-modified MWNTs (cMWCNTs), respectively. SSA is short for specific surface area.
